# Five primary melanomas in situ in a patient with recent tanning bed use, melanotan exposure, and anabolic hormone use

**DOI:** 10.1016/j.jdcr.2026.05.009

**Published:** 2026-05-12

**Authors:** Douglas Jaxon Vadner, Sidney Smith

**Affiliations:** a Department of Medical Education, Chicago Medical School at Rosalind Franklin University of Medicine and Science, North Chicago, Illinois; b Department of Dermatology, Georgia Skin and Cancer Clinic, Savannah, Georgia

**Keywords:** dysplastic nevi, melanocortin analog, melanoma in situ, melanotan, multiple primary melanoma, tanning bed exposure

## Introduction

Cutaneous melanoma incidence continues to rise worldwide.^1^ Concurrently, unregulated synthetic melanocortin analogs, including melanotan I and II, remain in use despite lack of regulatory approval. These agents are α-melanocyte–stimulating hormone analogs that promote melanogenesis via melanocortin receptor activation.^2^ Melanotan I primarily targets MC1R, whereas melanotan II is a nonselective agonist with broader systemic effects including sexual dysfunction and appetite suppression.^3^ Reported cutaneous effects include diffuse hyperpigmentation, darkening of preexisting nevi, and the development of new melanocytic lesions.

We present a case of multiple primary melanomas in situ arising in preexisting nevi that underwent reported darkening following melanotan exposure.

## Case presentation

A 49-year-old White male with Fitzpatrick skin phototype II presented for evaluation of multiple clinically atypical pigmented lesions on the trunk.

In mid-December 2025, the patient initiated tanning bed use, completing approximately 12 sessions over several weeks. Simultaneously, he began self-administering melanotan II (MT2), obtained through an online vendor, for cosmetic tanning purposes. He reported daily injections with dose escalation over approximately 1 week from 250 mcg to 500 mcg to 1000 mcg, with each dose level maintained for approximately 3 days. Following this, he transitioned to melanotan I (MT1) using a similar dosing and titration schedule.

Within approximately 1 week of initiating melanotan, he noted rapid darkening of multiple preexisting nevi, describing that several lesions “turned black.” He denied the development of new lesions but reported a localized hypopigmented patch on the hip during this period.

Although he was unable to estimate his lifetime tanning bed exposure, he reported intermittent use over several years, with most recent sessions occurring around the time of melanotan initiation. He also endorsed 2 prior blistering sunburns during adolescence and early adulthood.

After approximately 2-3 weeks of combined melanotan exposure and tanning bed use, he presented for evaluation of multiple clinically atypical pigmented lesions, and biopsies were performed on January 7, 2026.

His medical history was further notable for testosterone replacement therapy (testosterone cypionate), initiated approximately 2-3 years prior, obtained through an unregulated online source, dosed at 200 mg every 14 days since initiation. Family history was notable for melanoma in his paternal grandmother, with no additional family history of melanoma, pancreatic cancer, or other malignancy reported. The patient had not undergone prior genetic testing or routine skin examinations.

**Fig 1:**
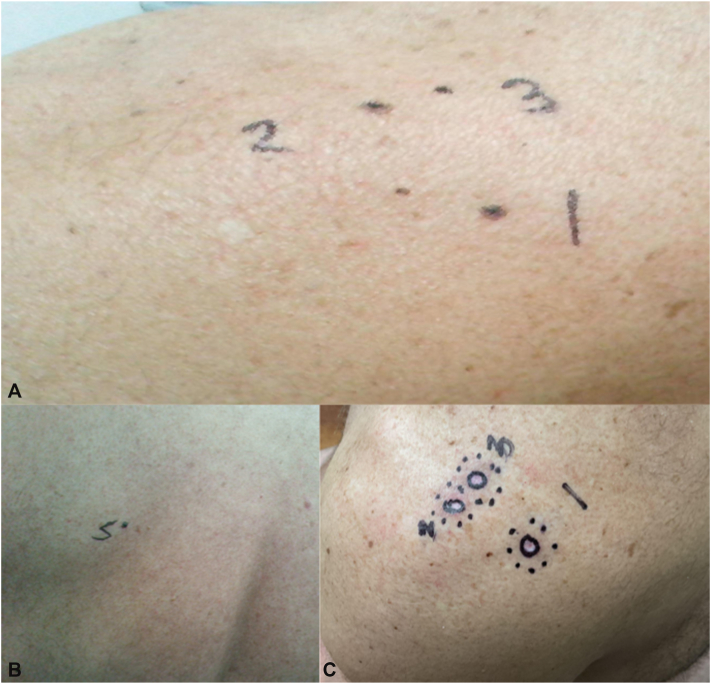
Representative clinically atypical pigmented lesions. **A,** Clinical overview of the upper left shoulder prior to biopsy demonstrating a high burden of melanocytic nevi, including multiple clinically atypical pigmented lesions. **B,** Close-up view of a clinically atypical pigmented lesion on the mid back prior to biopsy, demonstrating asymmetry, irregular borders, and variegated pigmentation. **C,** Clinical appearance of a lesion on the upper left shoulder following diagnostic biopsy, demonstrating biopsy site changes prior to definitive surgical excision.

Cutaneous examination revealed a high burden of melanocytic nevi across the trunk, several with clinical atypia. Five lesions on the left superior scapula, left medial posterior shoulder, left lateral posterior shoulder, right posterior shoulder, and left superior flank demonstrated asymmetry, irregular borders, and color variegation (Fig 1) and were selected for biopsy based on these features and recent change. Although numerous additional nevi were present, only the 5 most clinically atypical lesions were biopsied. Dermoscopy was not performed, as the lesions demonstrated concerning clinical features warranting biopsy.

**Fig 2:**
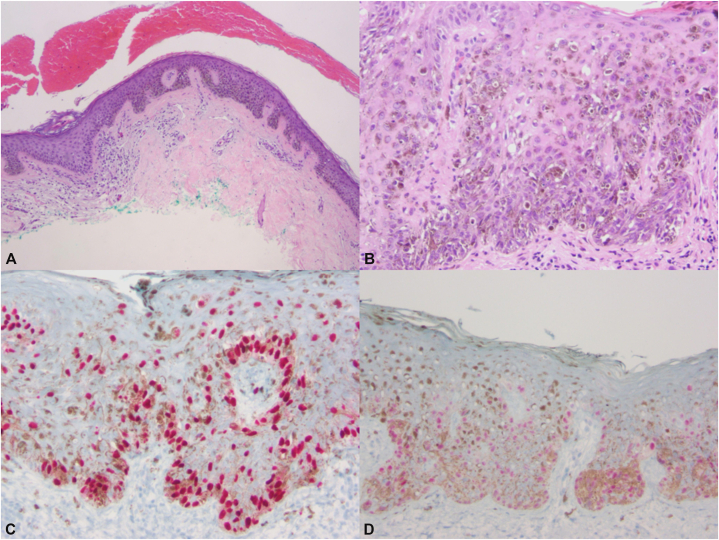
Histopathologic and immunohistochemical findings. **A,** Low-power hematoxylin–eosin (H&E) view demonstrating melanoma in situ arising in a severely dysplastic nevus. **B,** High-power H&E view showing atypical junctional melanocytes with nuclear enlargement and cytologic atypia. **C,** SOX-10 immunostain highlighting junctional melanocytic proliferation. **D,** PRAME immunohistochemistry demonstrating nuclear expression in a subset of atypical melanocytes.

Histopathologic examination demonstrated melanoma in situ arising in severely dysplastic nevi in all 5 specimens. Microscopic features showed atypical junctional melanocytes with cytologic atypia. Immunohistochemical staining with SOX-10 highlighted junctional melanocytes, and PRAME immunoreactivity was detected in a subset of atypical melanocytes. Complete surgical excision with appropriate margins was performed for all 5 melanoma in situ lesions. Findings are highlighted in Fig 2.

## Discussion

This case highlights multiple primary melanomas in situ in a patient with a high burden of atypical nevi, with rapid darkening of nevi within 1 week of melanotan initiation. This aligns with previously described melanocytic stimulation associated with melanotan exposure.

Melanotan compounds stimulate melanocortin receptors and have been associated with diffuse hyperpigmentation and nevus darkening.^4^ While case reports describe melanoma in exposed individuals, causality remains unproven.^4^^,^^5^ The brief duration of exposure further highlights uncertainty regarding dose–response relationships and latency in melanocytic neoplasia. Given the short interval between melanotan exposure and diagnosis, it is possible that these melanomas were preexisting and became clinically apparent due to pigmentary changes rather than arising de novo within this time frame.

**Fig 3:**
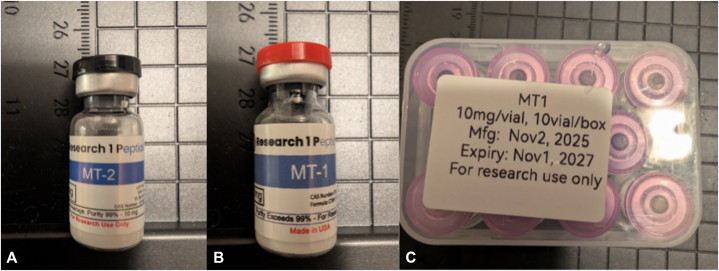
Patient-provided images documenting prior melanotan products used for injection. **A,** MT2 vial labeled “for research use only.” **B,** MT1 vial. **C,** MT1 packaging displaying concentration and labeling information.

Patient-provided images of melanotan I and II products are shown (Fig 3).

The patient also reported testosterone replacement therapy, representing an additional potential confounder. Although no association can be inferred, multiple exposures with potential biologic effects emphasize the multifactorial nature of melanoma risk. Bodybuilding behaviors and image-related goals may additionally involve increased ultraviolet exposure, representing an independent risk factor. Importantly, the patient’s history of frequent tanning bed use over a short interval represents a well-established and likely significant independent risk factor for melanoma, serving as a major confounding variable in interpreting any potential contribution of melanotan exposure.

Beyond the individual patient, this case is notable in the context of a re-emerging public health concern. Content related to melanotan use is widely disseminated across TikTok, Reddit, and Discord, where non-clinicians commonly provide instructions regarding dosing regimens, injection techniques, and personal experiences, often presented in a manner that resembles medical guidance.^6^ An analysis of TikTok content related to pro-tanning trends found that approximately 3% of videos referenced melanotan II use, including nasal spray and injectable formulations, highlighting the presence of unregulated melanogenic agents within social media tanning discourse.^7^ Regulatory agencies, including the Therapeutic Goods Administration in Australia, have issued public warnings advising against the use of melanotan-containing tanning products, citing unapproved status and potential serious health risks, including skin cancer.^8^ This resurgence underscores the importance of clinician awareness and patient counseling regarding unregulated tanning agents.

Several limitations should be acknowledged. A high burden of clinically atypical nevi raises consideration for dysplastic nevus syndrome; however, the absence of a strong family history (limited to a single melanoma in a grandparent) lowers suspicion, and additional lesions were not biopsied. Exposure history was based on patient self-report, and independent verification of compound composition, dosing, and purity was not available. Causality between melanotan exposure and melanoma development cannot be established from a single observational case.

This case highlights multiple primary melanomas in situ in preexisting nevi following melanotan exposure. Although causality cannot be established, clinicians should remain aware of the potential risks associated with unregulated melanogenic agents. Careful skin examination, patient education, and close surveillance may be warranted in individuals reporting prior use of these compounds.

## Conflicts of interest

None disclosed.
